# A Double Whammy: A Case Report and Review of the Literature on Pneumococcal Coinfection in COVID-19 Patients

**DOI:** 10.7759/cureus.13322

**Published:** 2021-02-13

**Authors:** Lubaina Ibrahim, Lokesh Manjani, Ahmad Nassar, Aleena R Mahmood, Hussam Ammar

**Affiliations:** 1 Medicine, MedStar Washington Hospital Center, Washington, DC, USA; 2 Internal Medicine, Medstar Washington Hospital Center, Washington, DC, USA

**Keywords:** covid-19, streptococcus pneumoniae, coinfection, pneumococcal infection, pneumonia

## Abstract

Bacterial coinfections which are infections present early in the course of coronavirus disease 2019 (COVID-19) infections before hospitalizations during the 2020 pandemic are rare. A 66-year-old male presented to the ED with a two-week history of subjective fever, dyspnea, and productive cough. He was diagnosed with coinfection of severe COVID-19 pneumonia and pneumococcal infection. He recovered from both infections and was discharged home. This report presents the features of this case and reviews the literature of similar cases of coinfection of COVID-19 pneumonia and pneumococcal infection.

## Introduction

Bacterial coinfections which are infections present early in the course of respiratory viral infections before hospitalizations are a leading cause of severe morbidity and mortality, especially among high risk groups such as the elderly [1]. Recent re-analysis of post-mortem lung cultures from 1918 influenza pandemic showed evidence of bacterial infection in >90% of specimens [1]. The rate of bacterial coinfection during the 2020 COVID-19 pandemic is lower than the rate of bacterial coinfection during the 1918 influenza H1N1 and 2009 influenza H1N1 pandemics [1-2]. This report presents a rare case of coinfection of COVID-19 pneumonia and pneumococcal infection in a 66-year-old man. The report also reviews the clinical features, radiological findings, and outcomes of cases of coinfection of COVID-19 pneumonia and pneumococcal infection in the English literature.

## Case presentation

**Figure 1:**
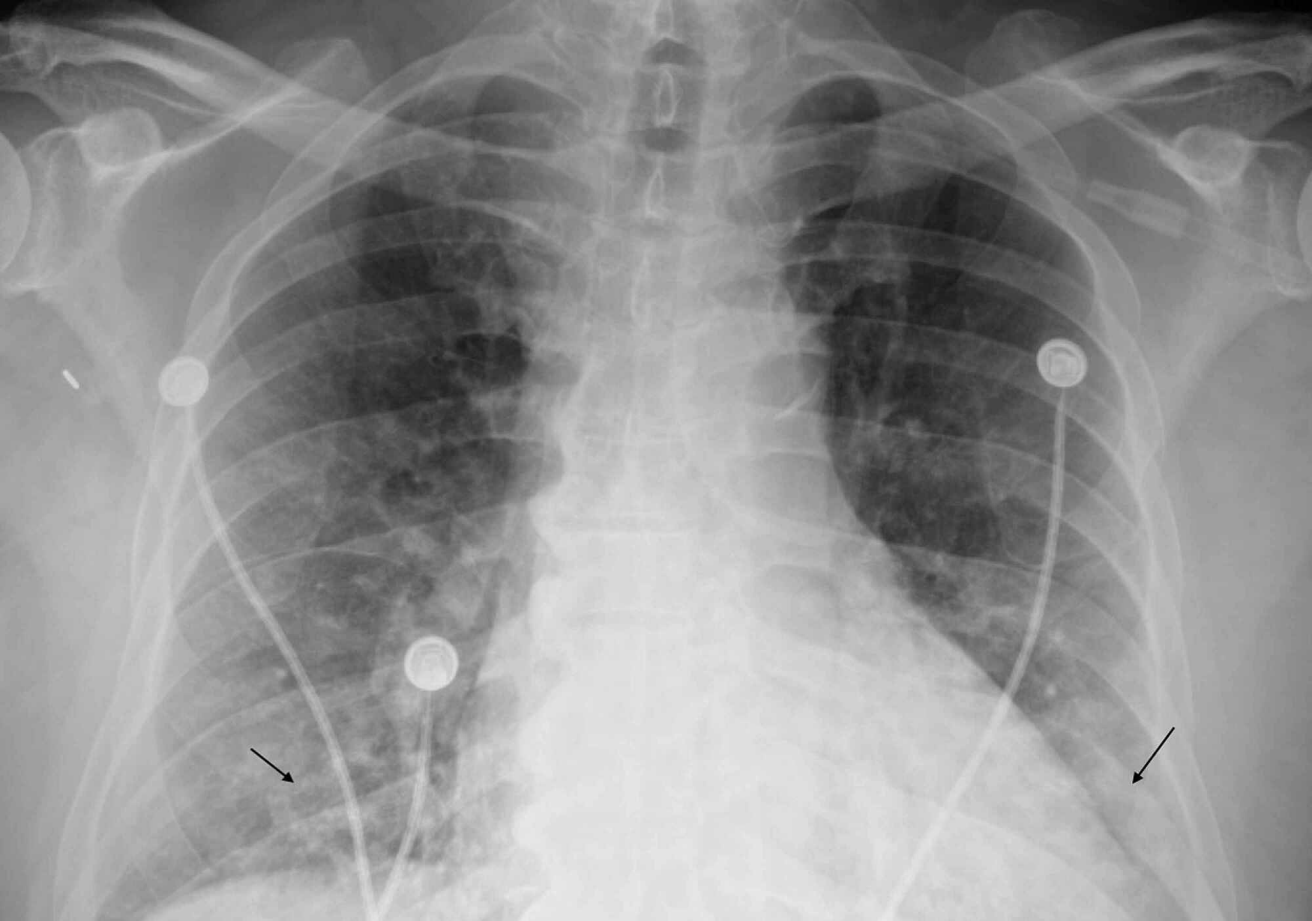
Chest radiography. Bilateral patchy basal infiltrates "arrows"  on admission CXR CXR, chest X-ray

A 66-year-old male presented to the ED with a two-week history of subjective fever, dyspnea, and a productive cough of rusty sputum. The symptoms started insidiously and improved after a few days of onset only to progressively worsen, bringing the patient to the hospital. The patient has a past medical history of diabetes mellitus (DM), chronic kidney disease (CKD), hypertension (HTN), coronary artery disease, and heart failure with preserved ejection fraction. On initial exam, he was tachypneic with a respiratory rate of 32 breaths/min, febrile with a temperature of 39.7℃, pulse rate of 101 beats/min, and blood pressure of 146/84 mmHg, and he required 15 L of oxygen/min through a nonrebreather mask to maintain his oxygen saturation above 94%. Lung auscultation was significant for crackles at lung bases bilaterally. Chest radiograph at presentation showed bilateral patchy basal infiltrates (Figure 1).

Laboratory work was significant for a creatinine level of 1.76 mg/dL, and the white blood cell count was 6,300 cells per cubic per millimeter with an absolute lymphocyte count of 1,800 cells per cubic millimeter, C-reactive protein of 143 mg/L, fibrinogen of 1026 mg/dL, D-dimer of 11.6 mcg/mL, and ferritin of 1071 ng/mL. Nasal swab for COVID-19 polymerase chain reaction (PCR) was positive. The patient was admitted to general medicine service; he received a loading dose of IV remdesivir 200 mg followed by 100 mg IV daily for five days. IV dexamethasone 6 mg daily was given for 10 days.

**Figure 2:**
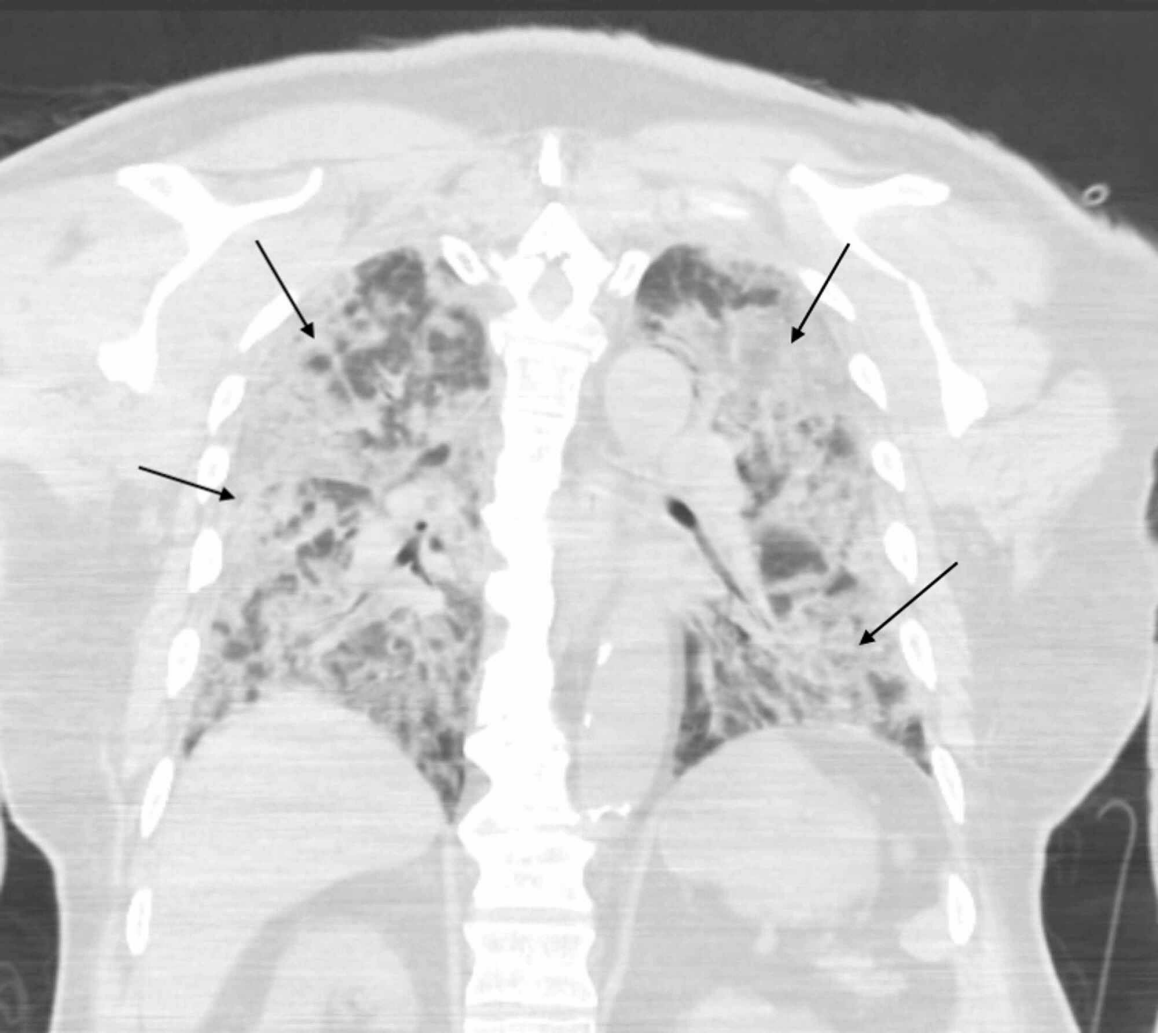
Chest CT scan. Bilateral consolidative infiltrates " arrows " on a follow up CT chest 48 hours after admission

The admitting physician suspected cobacterial infection because of the history of rusty sputum and administered ceftriaxone 2 g IV for five days upon admission to general medicine. Urine *Streptococcal pneumoniae* antigen was reported to be positive a few hours after his admission. Blood culture revealed no growth, and chest CT performed two days after admission revealed bilateral consolidative infiltrates (Figure 2).

Respiratory status improved slowly; he required 10 L of oxygen/min on day 5 and 5 L of oxygen/min on day 7 when he developed severe chest pain, which improved with nitroglycerine. An electrocardiogram revealed subtle ST segment depression in lead 3, and high sensitivity troponin I was elevated at 33.048 ng/L. An echocardiogram revealed an ejection fraction of 45% and hypokinetic anterior and lateral walls. Non-ST segment elevation myocardial infarction was diagnosed and managed conservatively with aspirin, clopidogrel, metoprolol, heparin, and atorvastatin with a plan for heart catheterization a few weeks after he recovered from his illness. The patient also developed acute kidney injury with a peak creatinine of 2.8 mg/dL, which recovered on conservative management to a baseline creatinine of 1.5 mg/dL. He also received a few doses of furosemide. He did not require intensive care admission, and his oxygen saturation was 98% on ambient air on day 10 but dropped to the low eighties with ambulation. He was discharged home on day 12 with home oxygen to be used with ambulation. Our patient had a follow up in the medicine clinic two weeks after his discharge, and he was very pleased that he recovered from COVID-19.

## Discussion

Respiratory viral infections predispose patients to bacterial coinfections, which lead to increased severity and mortality [1]. Bacterial pneumonia caused the majority of deaths during the 1918-1919 influenza pandemic [1]. The rate of bacterial coinfection in hospitalized influenza patients during the 2009 H1N1 influenza pandemic was 19% [3]. A recent review and meta-analysis that included 24 studies and defined coinfection as infection present at presentation and secondary infection as infection acquired during hospital stay, reported a rate of 3.5% for coinfection and a rate of 14.3% for secondary infection in July 2020 [2]. The current rate as of December 2020 is 4.6% and 16% for coinfection and secondary infection, respectively [4]. Mycoplasma species, Hemophilus influenza, and Pseudomonas infection were the most common bacterial isolates. Case reports and case series were excluded from this meta-analysis [2, 4]. *Streptococcus pneumoniae* is identified in 10%-15% of cases of community-acquired pneumonia [5].

Our literature search found 19 cases (Table 1) with a description of cases of coinfection with *S. pneumoniae* and COVID-19 that were published in four reports from Spain, the UK, and the USA [6-9]. We excluded two patients from the Spanish report, as the diagnosis of COVID-19 was based on radiological diagnosis and never confirmed with PCR [7].

**Table 1:** Cases of pneumococcal coinfection in COVID-19 patients. HTN, hypertension; DM, diabetes mellitus; CKD, chronic kidney disease; OSA, obstructive sleep apnea; COPD, chronic obstructive pulmonary disease

Study	Age	Symptoms on presentation	Comorbidities	Radiological findings	Diagnosis of Streptococcus pneumoniae	Length of stay	Outcome
Toombs et al. [6] UK	86	Dyspnea, fever, fatigue	Alzheimer's disease, HTN, and disseminated malignancy	Bilateral interstitial infiltrate	Blood culture	15 days	Death
Toombs et al. [6] UK	82	Chest pain, fever, and cough	DM, ischemic cardiomyopathy, HTN, and CKD	Bilateral patchy consolidation	Blood culture	21 days	Discharged
Cucchiari et al. [7] Spain	86	Fever, cough, dyspnea	DM, HTN, ischemic cardiomyopathy, CKD, aortic stenosis	Unilateral consolidative infiltrates	Pneumococcal urine antigen	NA	Discharged
Cucchiari et al. [7] Spain	65	Fever, cough, dyspnea, diarrhea	Depression	Bilateral interstitial infiltrate	Pneumococcal urine antigen	NA	Discharged
Cucchiari et al. [7] Spain	44	Fever, Cough, Hyposmia Dysgeusia Arthromyalgia	DM, Obesity, asthma	Bilateral interstitial infiltrates	Pneumococcal urine antigen	NA	Discharged
Rodriguez-Nava et al. [9], USA	92	Fever, dyspnea, myalgia, altered mental status	HTN, DM, hyperlipidemia	Unilateral infiltrates	NA	5 days	Discharged
Rodriguez-Nava et al. [9], USA	82	Cardiac arrest	HTN, DM, atrial fibrillation, asthma, dementia	Bilateral infiltrate	NA	7 days	Death
Rodriguez-Nava et al. [9], USA	75	Dyspnea, altered mental status	Stroke, OSA, seizure, hyperlipidemia	Unilateral infiltrate	NA	7 days	Death
Rodriguez-Nava et al. [9], USA	77	Dyspnea, lethargy	HTN, deep venous thrombosis, COPD, supraventricular tachycardia	Bilateral infiltrates	NA	2 days	Death
Rodriguez-Nava et al. [9], USA	81	Dyspnea, fever, cough, chills	DM, COPD-asthma, hyperlipidemia	Bilateral infiltrates	NA	6 days	Death
Rodriguez-Nava et al. [9], USA	74	Dyspnea, lethargy	HTN, dementia	Bilateral infiltrates	NA	19 days	Death
Rodriguez-Nava et al. [9], USA	90	Fatigue	HTN, DM	Unilateral infiltrate	NA	7 days	Discharged
Rodriguez-Nava et al. [9], USA	74	Fever, dyspnea, altered mental status, fatigue	HTN, Parkinson, hypothyroidism	Bilateral infiltrates	NA	8 days	Death
Rodriguez-Nava et al. [9], USA	72	Dyspnea, fever, lethargy	COPD, dementia, HTN	Unilateral infiltrates	NA	6 days	Death
Rodriguez-Nava et al. [9], USA	82	Altered mental status, anorexia	HTN, dementia	Unilateral infiltrates	NA	6 days	Death
Rodriguez-Nava et al. [9], USA	74	Dyspnea, cough, and fever	DM, COPD, stroke, HTN	Bilateral infiltrates	NA	21 days	Discharged
Pal et al. [8] USA	51	Alcohol intoxication, right pleuritic chest pain	Emphysema, human immune deficiency virus infection, opioid abuse	CT scan bilateral ground glass and right middle lobe consolidation	Sputum culture	8 days	Discharged
Pal et al. [8] USA	62	Dyspnea, dry cough, and pleuritic chest pain	DM, HTN, coronary artery disease	Bilateral lower lobe infiltrates	Pneumococcal urine antigen	2 days	Discharged
Pal et al. [8] USA	21	Pleuritic chest pain, fever, cough	DM	Bilateral perihilar infiltrates	Blood cultures	7 days	Discharged

The patient in this report was 66 years old, the median age for cases in our literature review was 75 years, and the ages ranged from 21 to 92. Four patients (21%) presented with fever, cough and dyspnea, similar to our patient. There was no description of the quality of cough as described in our report except in one patient who had a dry cough. The rusty sputum in this case was the single finding that prompted the initiation of antibiotics, and it has historically been thought to be a classic feature of pneumococcal pneumonia [10]. One study showed that rusty sputum has a positive predictive value of 23% for positive Gram staining and culture; S. pneumoniae did not grow in any patient with rusty sputum but grew from yellow green and green and creamy sputum samples [10]. Productive cough is not a unique finding for bacterial pneumonia, as it was present in approximately 50% of patients with COVID-19 in one report [11].

Fever and dyspnea were the most common symptoms identified from the literature review, and each was present in 11 patients (58%), while chest pain and altered mental status were present in four patients (21%). The median number of comorbidities for the patients in the literature search was three, and the patient presented in this case had five comorbidities. Hypertension was the most common comorbidity and was found in 13 (68%) patients, while diabetes was the second most common comorbidity and was present in 11 (52.6%) patients.

Bilateral infiltrates were the most common radiological findings in this series and were present in 13 (68%) patients. Unilateral infiltrates were present in six (31.5%) patients. A detailed description of the type of infiltrates was present in only seven cases: one case with lower lobe infiltrates, another case with perihilar infiltrates, three cases with bilateral interstitial infiltrates, and two cases with consolidative infiltrates. The patient in this case presented initially with bilateral patchy basal infiltrates that progressed in two days to bilateral consolidative infiltrates on chest CT. These findings overlap with the radiological findings of pneumococcal pneumonia, and the classic radiologic feature of S. pneumoniae is a consolidative lobar disease [12]. In one study of 40 patients with S. pneumoniae, 12 patients had a consolidative appearance, 12 patients had a patchy “bronchopneumonia” pattern, 9 patients had an interstitial pattern, and 7 patients had a mixed appearance [12]. Nine patients (47%) died in this series, and the overall 30-day mortality rate of hospitalized pneumococcal community-acquired pneumonia was 8% [13]. The mortality rate of hospitalized COVID-19 patients dropped from 25.6% in March to 7.6% in August 2020 [14].

## Conclusions

*Streptococcus pneumoniae* coinfection should be considered in patients with COVID-19 pneumonia with multiple comorbidities and advanced age. There are no published North American guidelines on the role of empiric antibiotics in patients with COVID-19 pneumonia. Patients should be evaluated on individual basis for the possibility of bacterial coinfection, and if antibiotics are to be initiated, re-evaluation in two to three days is needed to discontinue or adjust antibiotics based on the case progress and results of microbiological tests.
